# Elevated soluble CD226 in Takayasu arteritis is useful for differentiation from giant cell arteritis, disease activity assessment, and prognosis prediction

**DOI:** 10.1097/MD.0000000000042844

**Published:** 2025-06-20

**Authors:** Miki Nakano, Masahiro Ayano, Shoichi Fukui, Nozomi Iwanaga, Tomofumi Tatsutani, Ayako Takaki-Kuwahara, Yasutaka Kimoto, Mitsuteru Akahoshi, Kiyoshi Migita, Atsushi Kawakami, Yoshifumi Tada, Hiroaki Niiro

**Affiliations:** a Department of Medicine and Biosystemic Science, Graduate School of Medical Sciences, Kyushu University, Fukuoka, Japan; b Department of Immunology and Rheumatology, Division of Advanced Preventive Medical Sciences, Nagasaki University Graduate School of Biomedical Sciences, Nagasaki, Japan; c Department of Rheumatology, NHO Nagasaki Medical Center, Nagasaki, Japan; d Department of Rheumatology, Faculty of Medicine, Saga University, Saga, Japan; e Department of Rheumatology, Fukushima Medical University School of Medicine, Fukushima, Japan; f Department of Medical Education, Graduate School of Medical Sciences, Kyushu University, Fukuoka, Japan.

**Keywords:** biomarkers, disease activity, giant cell arteritis, soluble CD226, Takayasu arteritis

## Abstract

Takayasu arteritis (TAK) is characterized by vascular injury, in which endothelial cells and immune cells including natural killer cells, have key roles. CD226 is an activating receptor expressed on natural killer cells and T cells, and the soluble form of CD226 (sCD226) is increased in diseases involving these cells. Therefore, we investigated the utility of serum sCD226 as a biomarker for TAK. Serum sCD226 levels were measured using an enzyme-linked immunosorbent assay in 34 TAK patients and 21 giant cell arteritis (GCA) patients. The associations between sCD226 levels and the angiographic classification, disease activity, and prognosis of TAK were analyzed. Serum sCD226 levels were significantly higher in TAK patients than in GCA patients. In patients with TAK, serum sCD226 levels were significantly elevated in the group of type Ⅴ compared with type Ⅰ to Ⅳ. Serum sCD226 levels were also elevated in patients with active TAK and in those with poor responses to corticosteroids. Moreover, the cumulative probability of relapse was increased in patients with high sCD226 levels. Serum sCD226 levels differentiated TAK from GCA and were associated with disease activity and relapse of TAK. Thus, serum sCD226 might be a useful biomarker for the management of TAK.

## 1. Introduction

Takayasu arteritis (TAK) and giant cell arteritis (GCA) are defined as large vessel vasculitis (LVV).^[1]^ Although TAK and GCA are classified as different diseases based on age, clinical symptoms, and vascular imaging,^[2,3]^ they share some similarities, including constitutional symptoms, headache, ischemic manifestations, and elevated erythrocyte sedimentation rate and C-reactive protein levels.^[4,5]^ Although the onset of TAK was thought to be younger than that of GCA, late-onset TAK has been recognized recently.^[5,6]^ These things often make it difficult to distinguish between TAK and GCA. Furthermore, the management of TAK is also challenging. Diagnosis of TAK can be difficult because of its nonspecific symptoms in the early phase^[7]^ and the lack of characteristic autoantibodies or biomarkers.^[5,8]^ As the disease progresses, TAK frequently relapses,^[5]^ often without adequate dose reduction of corticosteroids, leading to arterial stenosis and occlusion, resulting in life-threatening ischemic symptoms.^[9]^ Therefore, the correct and early diagnosis of TAK is essential, along with monitoring disease activity and predicting prognosis. However, proper biomarkers have not yet been established.^[5,10–12]^

Although the pathogenesis of TAK is not fully understood, it is characterized by vascular injury, in which endothelial cells and immune cells play key roles.^[5,12–14]^ Genetic factors are also involved in pathogenesis,^[5,12–14]^ and recent genome-wide association studies (GWAS) have revealed the importance of CD8^+^ T cells and natural killer (NK) cells.^[15]^ Because NK cells mainly influence vascular injury in TAK,^[12–14,16–18]^ they play a pivotal role in TAK.

CD226 is expressed on the surface of NK cells and T cells, acting as an activating receptor.^[19–22]^ The CD226 ligands CD155 and CD112 are expressed on endothelial cells and epithelial cells.^[20]^ CD226 is involved in various immune functions in NK cells and endothelial cells, and the interactions between these cells.^[19–23]^ The soluble form of CD226 (sCD226), which is shed from the membrane type of CD226 (mCD226) by a certain protease,^[24]^ has been reported to be a useful biomarker for cancers^[25,26]^ and acute graft-versus-host disease.^[24,27]^ In addition, increased serum sCD226 levels were accompanied by decreased mCD226 on peripheral blood mononuclear cells,^[25]^ NK cells, and CD8^+^ T cells and reflected disease activity,^[26]^ indicating that sCD226 may be a biomarker reflecting the immune response in diseases in which NK cells and CD8^+^ T cells play key roles. Therefore, sCD226 can be a valuable biomarker for TAK.

This study aimed to clarify the utility of sCD226 as a biomarker for TAK by measuring serum sCD226 levels via enzyme-linked immunosorbent assay (ELISA) in patients with TAK.

## 2. Materials and methods

### 2.1. Study population

We investigated 34 TAK and 21 GCA patients treated at Kyushu University Hospital, Saga University Hospital, Nagasaki University Hospital, and NHO Nagasaki Medical Center, between 2004 and 2020. Patients who met the 2022 American College of Rheumatology/European League Against Rheumatism classification criteria for TAK^[2]^ and GCA^[3]^ were enrolled in this study; those with infection or cancer at the time of serum sample collection were excluded. Some of these patients were treated with corticosteroids, immunosuppressive agents, and biological agents, either as monotherapy or in combination. We used data from 33 healthy controls (HCs) collected in our previous study.^[28]^

This study was approved by the ethics committees of Kyushu University Hospital (approval number 30-282 and 2019-481) and Nagasaki University Hospital (approval number 15072753) in accordance with the Helsinki Declaration. Some participants gave written informed consent, and others were selected by an opt-out strategy.

### 2.2. Data collection

The following information was obtained from the medical records of the patients: demographic data, clinical manifestations, laboratory findings, medications at baseline and after treatment, and relapse after treatment. Patients with TAK were classified into types Ⅰ to Ⅴ using contrast-enhanced computed tomography based on the angiographic classification of Hata et al.^[29]^ Disease activity of TAK was evaluated using the National Institutes of Health (NIH) criteria: with active TAK defined as having an NIH score of ≥2.^[30]^ Relapse of TAK was defined as the worsening of an NIH score of ≥2.^[30]^ We defined patients who achieved an inactive state only with corticosteroids as having a good response to corticosteroids and others as having a poor response. There were no missing data in our analysis.

### 2.3. Enzyme-linked immunosorbent assay

Serum sCD226 levels were measured using sandwich ELISA according to previous reports.^[24,28]^ Briefly, 96-well plates were coated with purified anti-human CD226 (DNAM-1) antibody (TX25; BioLegend, San Diego) (8 μg/mL, 100 μL/well) for 2 hours at room temperature. The plates were washed with washing buffer (0.05% Tween 20), and then blocked using a blocking buffer (1% BSA in PBS, 100 μL/well) for 2 hours at room temperature. After washing, recombinant human DNAM-1/CD226 Fc chimera protein (as a standard) (R&D Systems, Minneapolis) and serum samples were added at 100 μL/well and incubated overnight at 4 °C. The plates were washed and incubated with human DNAM-1/CD226 biotinylated antibody (R&D Systems) (0.6 μg/mL, 100 μL/well) for 1 hour at room temperature, and then washed. Streptavidin–horseradish peroxidase (R&D Systems) (1:200 in a washing buffer, 100 μL/well) was added and incubated for 30 minutes at room temperature. The plates were washed and reacted with the 3,3′,5,5′-tetramethylbenzidine substrate reagent set (BD Biosciences, San Jose) (100 μL/well) for 20 minutes at room temperature. The reaction was terminated using H_2_SO_4_ (2N) (50 μL/well). Absorbance was measured at 450 nm using a microtiter plate reader (Thermo Fisher Scientific, Waltham). All values were determined in duplicate. The assay range was 0.1 to 20.0 ng/mL.

### 2.4. Statistical analysis

The data are expressed as median and interquartile range unless otherwise stated. Differences between the 2 groups were evaluated using Student *t* test for normally distributed continuous variables or using the Mann–Whitney *U* test for non-normally distributed variables. The cumulative probability of relapse was analyzed using the Kaplan–Meier method and log-rank test. All tests were two-tailed, with *P*-values <.05 considered statistically significant. All analyses were performed using JMP software, version 17 (SAS Institute, Cary).

## 3. Results

### 3.1. Serum sCD226 levels are elevated in patients with TAK

**Figure 1.**
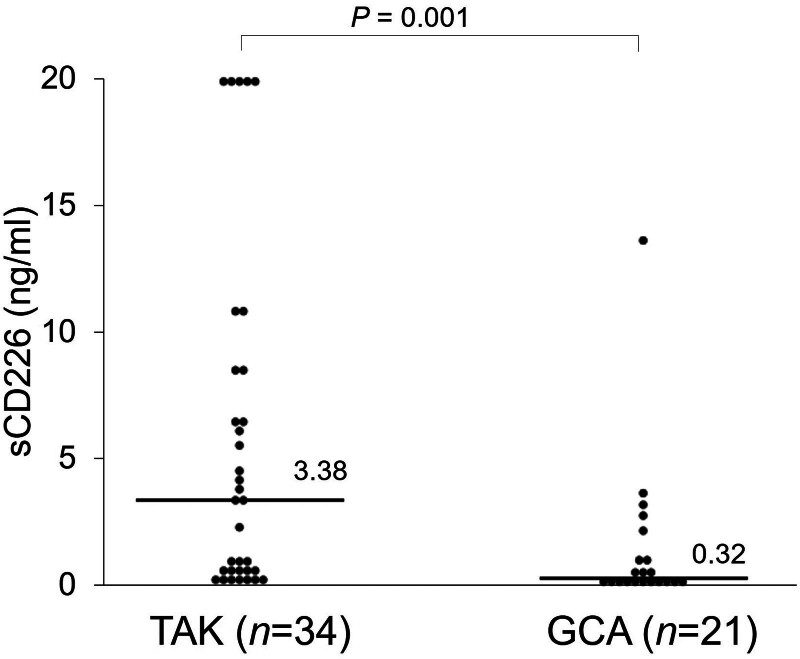
Serum sCD226 levels in patients with Takayasu arteritis and giant cell arteritis. Serum sCD226 levels were compared between patients with TAK and GCA. Each data point represents a single subject. The horizontal lines show the median. Statistical differences among groups were evaluated using the Mann–Whitney *U* test. GCA = giant cell arteritis, sCD226 = soluble CD226, TAK = Takayasu arteritis.

To investigate the association between sCD226 and LVV, serum sCD226 levels were measured using ELISA in 34 patients with TAK and 21 patients with GCA. Although no significant differences were observed between TAK and GCA patients in terms of gender, TAK patients were significantly younger than GCA patients because of the classification criteria.^[2,3]^ The baseline characteristics of patients with TAK and GCA are presented in Table 1. Serum sCD226 levels were significantly elevated in patients with TAK than in patients with GCA (3.38 ng/mL [0.36–8.50] vs 0.32 ng/mL [0.10–1.60]; *P* = .001) (Fig. 1). Although there was no significant difference, serum sCD226 levels were higher in 14 TAK patients than in 14 age- and gender-matched HCs (2.80 ng/mL [0.37–4.90] vs 0.48 ng/mL [0.13–1.42]; *P* = .12).

**Table 1:** Baseline characteristics of patients with Takayasu arteritis and giant cell arteritis.

Characteristics	TAK (n = 34)	GCA (n = 21)
Age, mean (S.D.), years	25.7 (9.1)	69.7 (7.3)
Female, *n* (%)	30 (88)	14 (67)
Disease duration, median [IQR], years	6.0 [1.0–53.0]	2.0 [1.0–2.0]
HLA-B52*, *n* (%)	15 (63)	4 (80)
*2022 ACR/EULAR classification criteria for TAK*		
Angina or ischemic cardiac pain, *n* (%)	13 (38)	
Arm or leg claudication, *n* (%)	6 (18)	
Vascular bruit, *n* (%)	20 (59)	
Reduced pulse in upper extremity, *n* (%)	8 (24)	
Carotid artery abnormality, *n* (%)	8 (24)	
Systolic blood pressure difference in arms ≥ 20 mm Hg, *n* (%)	13 (38)	
Number of affected arterial territories, *n* (%)		
1, 2, ≥3	4 (12), 9 (26), 21 (62)	
Symmetric involvement of paired arteries, *n* (%)	13 (54)	
Abdominal aorta involvement with renal or mesenteric involvement, *n* (%)	12 (41)	
*2022 ACR/EULAR classification criteria for GCA*		
Morning stiffness in shoulders/neck, *n* (%)		2 (10)
Sudden visual loss, *n* (%)		4 (19)
Jaw or tongue claudication, *n* (%)		9 (43)
New temporal headache, *n* (%)		14 (67)
Scalp tenderness, *n* (%)		6 (29)
Abnormal examination of the temporal artery, *n* (%)		12 (60)
Maximum ESR ≥ 50 mm/hour or maximum CRP ≥ 1.0 mg/dl, *n* (%)		20 (95)
Positive temporal artery biopsy or halo sign on temporal artery ultrasound, *n* (%)		6 (29)
Bilateral axillary involvement, *n* (%)		2 (10)
FDG-PET activity throughout aorta, *n* (%)		5 (24)
*Angiographic classification*, *n* (%)		
I	4 (12)	
II	18 (53)	
III	4 (12)	
IV	2 (5)	
V	6 (18)	
*TAK disease activity*		
Active (NIH score ≥ 2), *n* (%)	27 (80)	
NIH score, median [IQR]	3 [1–4]	
CRP, median [IQR], mg/dl	4.27 [0.35–8.37]	8.56 [2.86–13.77]
ESR, median [IQR], mm/hour	64 [22–101]	95 [54–116]
Drug-naive, *n* (%)	22 (65)	20 (95)
Corticosteroid use, *n* (%)	12 (35)	1 (5)
*Prednisolone equivalent dose, median* [*IQR*], *m*g/*day*	10 [5–17]	2
Immunosuppressive agent use, *n* (%)	7 (21)	0
Biological agent use, *n* (%)	5 (15)	0

ACR = American College of Rheumatology, CRP = C-reactive protein, ESR = erythrocyte sedimentation rate, EULAR = European League Against Rheumatism, GCA = giant cell arteritis, IQR = interquartile range, NIH = National Institutes of Health, TAK = Takayasu arteritis.

* HLA-B52 was measured in 24 TAK patients and 5 GCA patients.

### 3.2. Serum sCD226 levels are elevated in patients with active TAK and reflect disease activity

**Figure 2.**
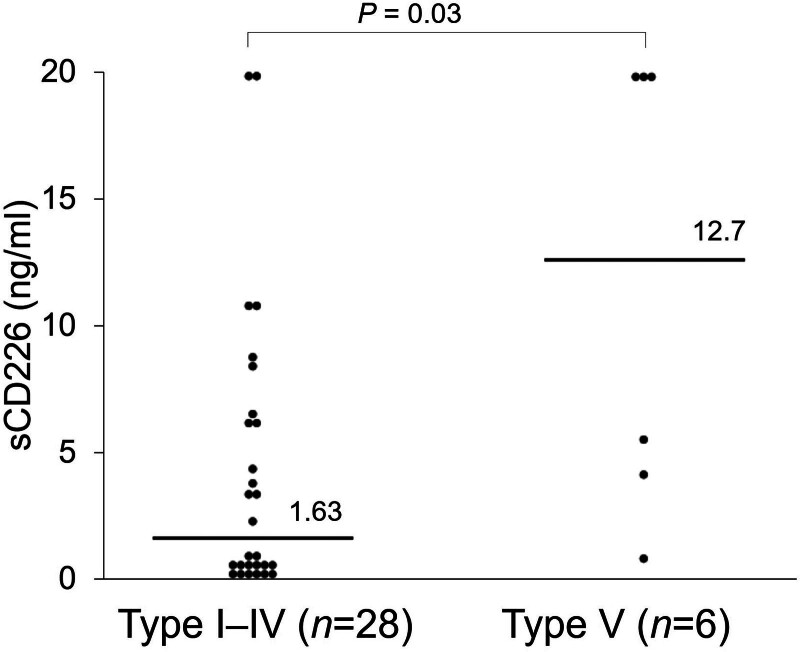
Serum sCD226 levels in patients with Takayasu arteritis. Serum sCD226 levels were compared between the group of type Ⅰ to Ⅳ and type Ⅴ in TAK patients. Each data point represents a single subject. The horizontal lines show the median. Statistical differences among groups were evaluated using the Mann–Whitney *U* test. sCD226 = soluble CD226, TAK = Takayasu arteritis.

To assess the association between sCD226 levels and the extent of vascular lesions, the relationship between serum sCD226 levels and the angiographic classification of TAK was evaluated.^[29]^ There were 6 patients in the group of type Ⅴ, which had the most extensive lesions, and 28 patients in the group of other types excluding type Ⅴ. Serum sCD226 levels were significantly increased in the group of type Ⅴ compared with type Ⅰ to Ⅳ (12.7 ng/mL [3.33–20.0] vs 1.63 ng/mL [0.32–6.39]; *P* = .03) with levels higher than 20 ng/mL in 3 out of 6 in the group of type Ⅴ compared to only 2 out of 28 in type Ⅰ to Ⅳ (Fig. 2).

**Figure 3.**
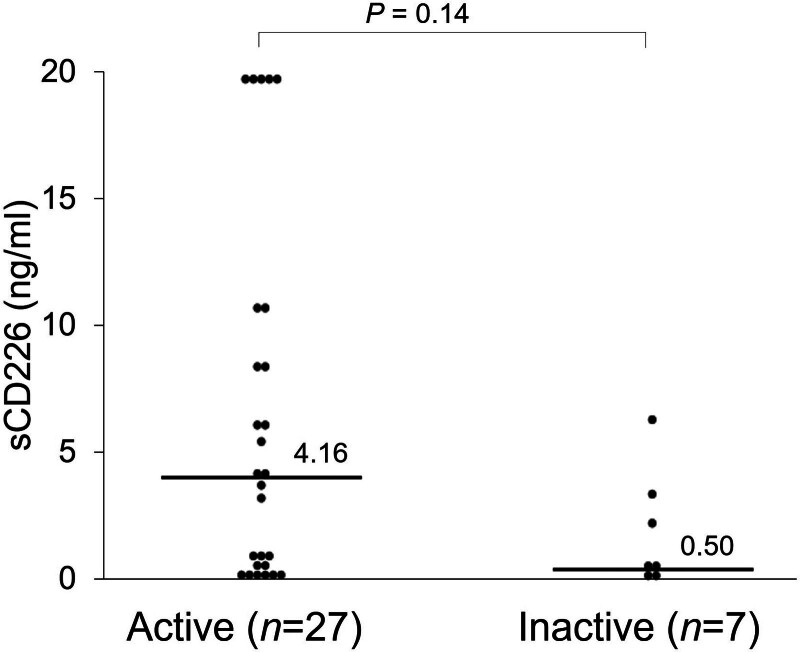
Serum sCD226 levels in patients with Takayasu arteritis. Serum sCD226 levels were compared between active and inactive TAK patients. Each data point represents a single subject. The horizontal lines show the median. Statistical differences among groups were evaluated using the Mann–Whitney *U* test. sCD226 = soluble CD226, TAK = Takayasu arteritis.

Next, the relationship between serum sCD226 levels and TAK disease activity was assessed. There were 27 active TAK patients and 7 inactive TAK patients. Serum sCD226 levels were elevated in patients with active TAK (4.16 ng/mL [0.38–10.86] vs 0.50 ng/mL [0.31–3.46]; *P* = .14) with levels higher than 20 ng/mL in 5 patients with active TAK compared to none with inactive TAK (Fig. 3).

**Figure 4.**
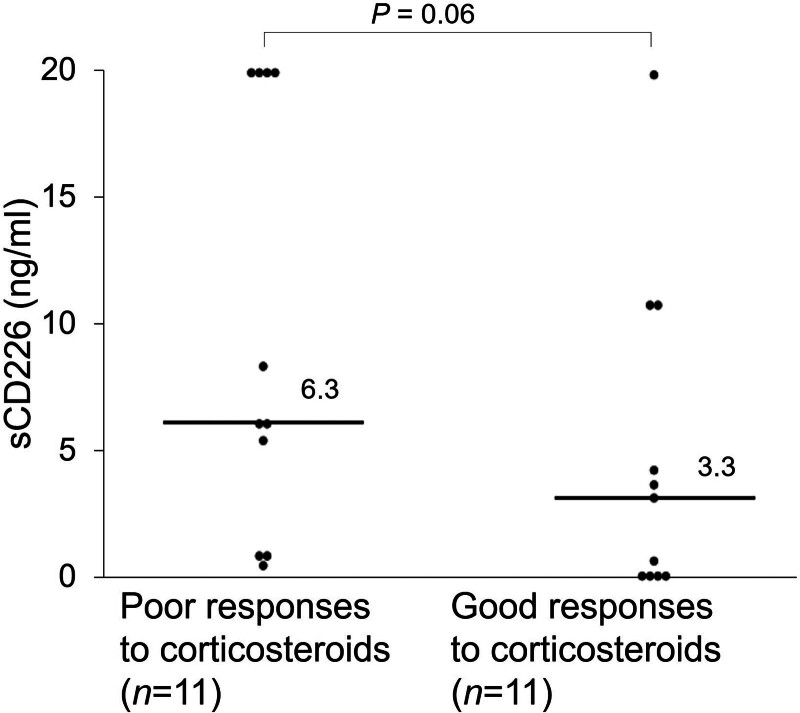
Serum sCD226 levels in drug-naive patients with active Takayasu arteritis. Serum sCD226 levels were compared between patients with good and poor responses to corticosteroids. Each data point represents a single subject. The horizontal lines show the median. Statistical differences among groups were evaluated using the Mann–Whitney *U* test. sCD226 = soluble CD226.

There were 22 drug-naive patients with active TAK in this study. To examine the relationship between sCD226 levels and response to therapy, we compared serum sCD226 levels between patients with good and poor responses to corticosteroids, revealing elevations in those with poor responses (3.30 ng/mL [0.25–10.86] vs 6.30 ng/mL [0.96–20.0]; *P* = .06) (Fig. 4).

### 3.3. Serum sCD226 levels can predict disease relapse

**Figure 5.**
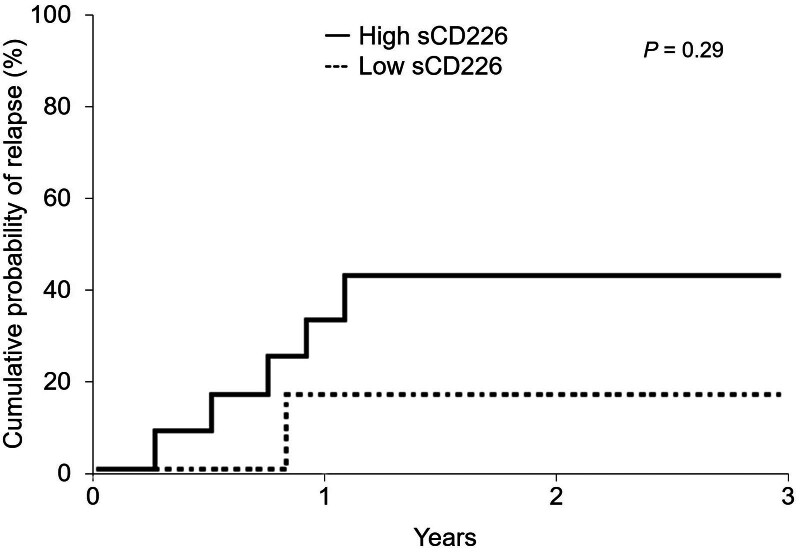
Kaplan–Meier analysis of the cumulative probability of relapse. Curves were compared between patients with TAK having high and low sCD226 levels using log-rank tests. sCD226 = soluble CD226, TAK = Takayasu arteritis.

Relapse after treatment was observed in 6 out of 22 drug-naive active TAK patients. We defined a median sCD226 level of 3.3 ng/mL among patients with TAK as the cutoff value to classify patients as having high or low sCD226 levels, and 14 patients had high sCD226 levels. Although there was no significant difference, the cumulative probability of relapse was higher in patients with high sCD226 levels (Fig. 5, shown in solid lines) than in those with low sCD226 levels (Fig. 5, shown in dashed lines) (*P* = .29).

## 4. Discussion

Our results revealed that serum sCD226 levels were higher in TAK patients than in GCA patients, significantly increased in active TAK patients, and associated with disease activity and prognosis.

In this study, we aimed to investigate the association between sCD226 and LVV and found that serum sCD226 levels were significantly elevated in patients with TAK compared with GCA and HCs. The findings indicate that sCD226 might serve as a useful biomarker to diagnose TAK, which is often difficult to diagnose early and differentiate from GCA.^[4–8]^ Previous reports on cancer patients showed an increase in serum sCD226 levels accompanied by a decrease in mCD226 on immune cells, such as NK cells.^[25,26]^ Regarding autoimmune diseases, our previous study demonstrated that serum sCD226 levels were increased in patients with active systemic lupus erythematosus (SLE),^[28]^ whereas Huang et al showed that expression of mCD226 on NK cells was decreased in patients with active SLE.^[31]^ These findings suggest the involvement of CD226 and the association of serum sCD226 levels and mCD226 on NK cells in SLE, although further analyses are needed. The present study showed that serum sCD226 levels were elevated in TAK patients but not in GCA patients. Despite their similarities in pathogenesis, TAK and GCA have key differences,^[4,14]^ particularly in their roles in NK cells.^[12–18]^ Our results might reflect alterations in NK cells, which are important in the pathogenesis of TAK. However, the present study did not examine immune cells, including NK cells; thus, performing cellular immunophenotyping and analyzing the relation between sCD226 and mCD226 levels would greatly enhance the scientific impact. The mechanism underlying the elevation of serum sCD226 levels in TAK also needs to be further studied in the future.

Our study showed that serum sCD226 levels were elevated in patients with active TAK and those with poor responses to corticosteroids and that the cumulative probability of relapse tended to be higher in patients with high sCD226 levels. Monitoring disease activity in patients with LVV is recommended by the 2018 European League Against Rheumatism recommendations^[10]^ and the 2021 American College of Rheumatology guidelines,^[11]^ and imaging modalities have been advanced recently.^[32]^ However, appropriate biomarkers have not yet been established.^[5,10–12]^ In our study, serum sCD226 levels were associated with disease activity and prognosis. Thus, sCD226 might be a useful biomarker for TAK.

Our results suggest an association between CD226 and TAK although the mechanism remains unknown. Other autoimmune diseases, such as SLE,^[28,31,33–37]^ rheumatoid arthritis,^[36,38–42]^ and systemic sclerosis,^[43–45]^ have been associated with CD226, specifically in terms of genetic factors and immune responses. Several GWAS have reported associations between the nonsynonymous rs763361 polymorphism in CD226 and SLE,^[33–36]^ rheumatoid arthritis,^[36,38–40]^ and systemic sclerosis.^[43,44]^ In addition, some studies on immune cells, including our group, have suggested the involvement of CD226 in autoimmune diseases.^[28,31,37,41,45]^ Regarding the association between CD226 and LVV, there are few GWAS and no immunological studies; only one Spanish study showed that CD226 gene variants are not involved in GCA,^[46]^ and the association between CD226 gene variants and TAK is not revealed. In our analysis, serum sCD226 levels were increased in the group of type Ⅴ, which had the most extensive lesions, indicating that sCD226 might reflect the extent of vascular lesions and affect endothelial cells as well. Given that mCD226 expression on CD8^+^ T cells was associated with endothelial injury^[45]^ and sCD226 could directly affect cells expressing the ligand, such as cancer cells,^[25,47]^ sCD226 might be involved in the pathogenesis of TAK. Based on this result, further GWAS and immunological studies are required.

Our study had several limitations. First, this study had a small sample size with only Japanese patients. Since the incidence of TAK varies according to ancestry,^[5,12]^ further studies should include a larger sample size and/or other ancestries. Second, there was some difference in the number of patients when comparing groups, with only a few patients in some groups, especially patients in the group of type Ⅴ and those with relapse. To overcome the limitations of comparative power, future validation studies are required to involve a sufficiently large number of patients in more centers. Third, the functions of sCD226 remain unknown, and these need to be clarified in further investigations. Finally, our study was retrospective. To ensure the association between sCD226 levels with the disease activity and prognosis of TAK, a prospective study with longitudinal assessments should be conducted.

In conclusion, serum sCD226 levels were elevated in patients with TAK and were associated with disease activity and disease relapse. Serum sCD226 might be a useful biomarker for managing TAK, and its monitoring can help in the precise management of TAK.

## Acknowledgments

The authors thank Enago for the English language review.

## Author contributions

**Conceptualization:** Miki Nakano, Masahiro Ayano.

**Data curation:** Miki Nakano, Masahiro Ayano.

**Formal analysis:** Miki Nakano, Masahiro Ayano.

**Funding acquisition:** Masahiro Ayano.

**Investigation:** Miki Nakano, Shoichi Fukui, Nozomi Iwanaga, Tomofumi Tatsutani, Ayako Takaki-Kuwahara, Yasutaka Kimoto, Mitsuteru Akahoshi, Kiyoshi Migita, Yoshifumi Tada.

**Project administration:** Miki Nakano, Masahiro Ayano.

**Resources:** Miki Nakano, Masahiro Ayano, Shoichi Fukui, Nozomi Iwanaga, Ayako Takaki-Kuwahara, Kiyoshi Migita, Yoshifumi Tada.

**Supervision:** Masahiro Ayano, Atsushi Kawakami, Hiroaki Niiro.

**Validation:** Miki Nakano, Tomofumi Tatsutani.

**Visualization:** Miki Nakano.

**Writing – original draft:** Miki Nakano.

**Writing – review & editing:** Miki Nakano, Masahiro Ayano, Shoichi Fukui, Nozomi Iwanaga, Tomofumi Tatsutani, Ayako Takaki-Kuwahara, Yasutaka Kimoto, Mitsuteru Akahoshi, Kiyoshi Migita, Atsushi Kawakami, Yoshifumi Tada, Hiroaki Niiro.
